# Follicular Epithelial Dysplasia as Hashimoto Thyroiditis-Related Atypia: a Series of 91 Specimens

**DOI:** 10.1007/s12022-021-09679-w

**Published:** 2021-05-15

**Authors:** Ivana Kholová, David Kalfert, Jarkko Lintusaari, Erja Rajakorpi, Marie Ludvíková

**Affiliations:** 1 Fimlab Laboratories, Tampere, Finland; 2 Faculty of Medicine and Health Technology, Tampere University, Tampere, Finland; 3 Department of Otorhinolaryngology and Head and Neck Surgery, First Faculty of Medicine, University Hospital Motol, Charles University, Prague, Czech Republic; 4 Pathology Department, Seinäjoki Central Hospital, Seinäjoki, Finland; 5 Department of Biology, Faculty of Medicine in Pilsen, Charles University, Pilsen, Czech Republic

**Keywords:** Follicular epithelial dysplasia, Hashimoto thyroiditis, Papillary thyroid carcinoma, Inflammation, Fine-needle aspiration, Cytohistologic discrepancy

## Abstract

Follicular epithelial dysplasia (FED) is described as Hashimoto thyroiditis-related atypia and is thought to be a possible precancerous lesion. Dysplasia as an interface between normal state and carcinoma is described in a wide range of diseases and carcinogenesis chains. On the other hand, inflammation-related atypia and cancerogenesis is also widely studied. In this study, we retrospectively analyzed 91 specimens of thyroid gland surgical resections with FED during a 10-year-period at the university hospital pathology department. The study population consisted of 68 females and 15 males aged between 22 and 86 years. The preoperative cytology diagnoses had mainly been in the indeterminate categories with prevailing AUS/FLUS results in the FED-only group (p = 0.005) and suspicious for malignancy and malignant in the group with FED plus adjacent malignancy. The decision for surgery was malignancy related in 48.2% of the cases. The lesions were sized 0.1–3.5 mm and multifocal in 45.1% of the cases. Immunohistochemically, the atypical cells were cyclin D1-positive in 67.5%, galectin-3 in 72.7%, CK19 in 85.7%, and HBME-1 in 87.0% of cases. In conclusion, FED is suggested to be a pathogenetic link between inflammation-related atypia and papillary carcinoma and thus a premalignant precursor of papillary carcinoma in HT as 36.1% of the specimens contained also papillary carcinoma in the present study. Both histopathological nuclear features and the immunoprofile of FED are widely shared with that of papillary carcinoma.

## Introduction

Hashimoto thyroiditis (HT) known also as chronic lymphocytic thyroiditis is an autoimmune disease microscopically featuring diffuse lymphoplasmacytic inflammation with lymphoid follicles containing germinal centres, follicular epithelial damage, epithelial atrophy, oncocytic and rarely squamous metaplasia and fibrosis [1].

A close relation of inflammation and cancer was noticed in many studies. Already in 1863, the father of modern pathology Rudolf Virchow suggested a link between inflammation and cancer observing inflammatory cells in neoplastic tissue [2, 3]. Moreover, the inflammation belongs to the enablers of malignancy and is involved in the pathogenesis of cancer as it was introduced by Hanahan and Weinberg in 2011 [4]. Tumor-promotion inflammation makes possible the acquisition of functional capabilities called hallmarks of cancer by supplying bioactive molecules to the tumor microenvironment. These molecules are responsible for cell growth, resisting cell death, inducing angiogenesis etc. [5]. Tumor-associated inflammatory cells such as dendritic cells, mast cells, macrophages, T-lymphocytes, regulatory T cells, and natural killer cells are observed in many tumors including thyroid carcinomas [2, 6]. The association of HT with thyroid cancer, namely, thyroid papillary carcinoma, is reported with variable frequency up to 38% [2, 6]. The pathogenetic mechanism is nevertheless under debate with at least the following possibilities: tumor immune-escape mechanism, inflammatory immune response to preexisting autoimmunity and cross-reacting antitumor immunity [7].

Inflammatory bowel disease–related chronic inflammation is well recognized as a risk factor for colorectal cancer development with epithelial changes including a variety of inflammation-induced hyperplasia to dysplasia as well [8]. Dysplasia, that is disordered growth, is characterized by both cellular and architectural changes at the interface between normal state and carcinoma, being described in a wide range of tissues and organs [9].

In parallel, HT is characterized by epithelial changes [1], and variable atypia related to HT has been observed [10–14]. The elegant paper by Chui et al. used the term of follicular epithelial dysplasia (FED) in the thyroid gland with HT for the first time [15]. The FED lesions were described as groups of atypical cells with a diameter less than 1.0 cm with mild to moderate nuclear enlargement, membrane irregularities, chromatin margination, grooves, and crowding of nuclei with optical clearings variably present. Architecturally, the foci are irregularly shaped forming follicles, trabeculae, nests, and solid areas, but no papillae. Those groups are distinct from surrounding epithelium as well as from papillary microcarcinoma by lacking infiltrative growth and stromal desmoplasia [15].

In the present study, we analyzed morphological, immunohistochemical, and clinical characteristics of FED in HT specimens collected in a tertiary care university centre during a 10-year period.

## Materials and Methods

The archives and consultation files of the Pathology Department, Fimlab Laboratories, Tampere, Finland, were searched for HT cases with atypia fulfilling the histopathological criteria of FED. The epidemiological, laboratory, histopathological, and immunohistochemical features of these cases were analyzed.

Immunohistochemical staining was done on 2-µm-thick sections with Ventana Life Sciences Benchmark XT© Staining module (Ventana Medical Systems, Inc., Tucson, Arizona (AZ), USA) using antibodies against cytokeratin 19 (CK19) (clone A53-B/A2.26; Ventana Medical Systems, Inc., Tucson, AZ, USA), galectin-3 (clone 9c4; Ventana Medical Systems, Inc., Tucson, AZ, USA), cyclin D1 (clone SP4, Ventana Medical Systems, Inc., Tucson, AZ, USA) and Hector Battifora mesothelial-1 (HBME-1) (clone HBME-1; Ventana Medical Systems, Inc., Tucson, AZ, USA). The FED lesion was assessed as positive if > 10% of cells were positive in agreement with a previous study [15].

All statistical analyses were performed using IBM SPSS statistics (version 22.0; SPSS, IBM, Armonk, NY, USA). The data were analyzed using Fisher’s exact test (two-tailed), Pearson’s chi-square test and Mann-Whitney U test. *P*-values < 0.05 were considered statistically significant in all statistical analyses.

## Results

The study cohort consisted of 91 samples from 83 patients operated on within a 10-year-period (2010–2019). In addition to Fimlab Laboratories archival cases, there were 5 consultation cases from regional pathology laboratories. There were 68 females and 15 males with the average age of 54.5 ± 10.8 years (ranged from 22 to 86 years). The specimens were total thyroidectomies in 37 (44.6%) cases and lobectomies in the rest of the cases. The reasons for surgery were clinical or fine-needle aspiration (FNA) diagnosed malignancy or suspicion, including lobectomies after malignancy in a contralateral lobe (n = 44 cases, 48.2%), goiter-related symptoms (n = 43, 47.4%), hyperthyroidism (n = 2, 2.2%) and hyperparathyroidism (n = 2, 2.2%). The average amount of blocks/specimen was 16 (range 6–55). In case of malignancy, the specimen was entirely blocked.

In addition to HT, histopathological diagnoses consisted of malignant neoplasms (n = 38 (45.8%), including papillary carcinoma (n = 30, microcarcinoma in 13 cases), follicular carcinoma (n = 6), medullary carcinoma (n = 1), and anaplastic carcinoma (n = 1)). Benign neoplasms (n = 9 (10.8%)) were all follicular adenomas. There were 2 cases with multiple tumors: one with a follicular carcinoma and a papillary microcarcinoma and the other with a papillary microcarcinoma and a follicular adenoma. The neoplastic cases are summarized in Table 1. There were 14 immunoglobulin G4 (IgG4)-positive HT cases (16.9%) [16], but there has not been shown any relationship between FED and IgG4 positivity.

**Table 1:** The summary of neoplasms, their subtypes and cases with multiple neoplasms in FED cases

Neoplasms (n = 47, 56.6%)	Malignant neoplasms (n = 38, 45.8%)				Benign neoplasms (n = 9, 10.8%)
Histological type	Papillary carcinoma	Follicular carcinoma	Medullary carcinoma	Anaplastic carcinoma	Follicular adenoma
n	30	6	1	1	9
Histological subtype	Papillary microcarcinoma	ND	ND	ND	ND
n	13	ND	ND	ND	ND
Case 1 with 2 neoplasms	1 papillary microcarcinoma	1 follicular carcinoma	-	-	-
Case 2 with 2 neoplasms	1 papillary microcarcinoma	-	-	-	1 follicular adenoma

In 53 (63.9%) cases, there was a preceding FNA taken. The cytopathological diagnoses according to the Bethesda System for Reporting Thyroid Cytopathology (TBSRTC) [17, 18] are summarized in Table 2. Strikingly, in both the malignant and the benign groups, the majority of cases (n = 20) were placed into the indeterminate categories. Even in the FED-only group, 8 cases (32%) were diagnosed as suspicious for malignancy or malignant. In the atypia of undetermined significance/follicular lesion of undetermined significance (AUS/FLUS) category, there were significantly more FED-only cases (12 vs. 3, *p* = 0.005).

**Table 2:** The summary of TBSRTC categories in preoperative FNA samples according to final pathological anatomical diagnosis (PAD)

SD (Bethesda categories)	PAD	
	FED	FED and carcinoma
Insufficient	2	1
Benign	2	2
AUS/FLUS	12	3
Follicular neoplasm	1	2
Suspicious for malignancy	7	15
Malignant	1	5
Total	25	28

**Fig. 1:**
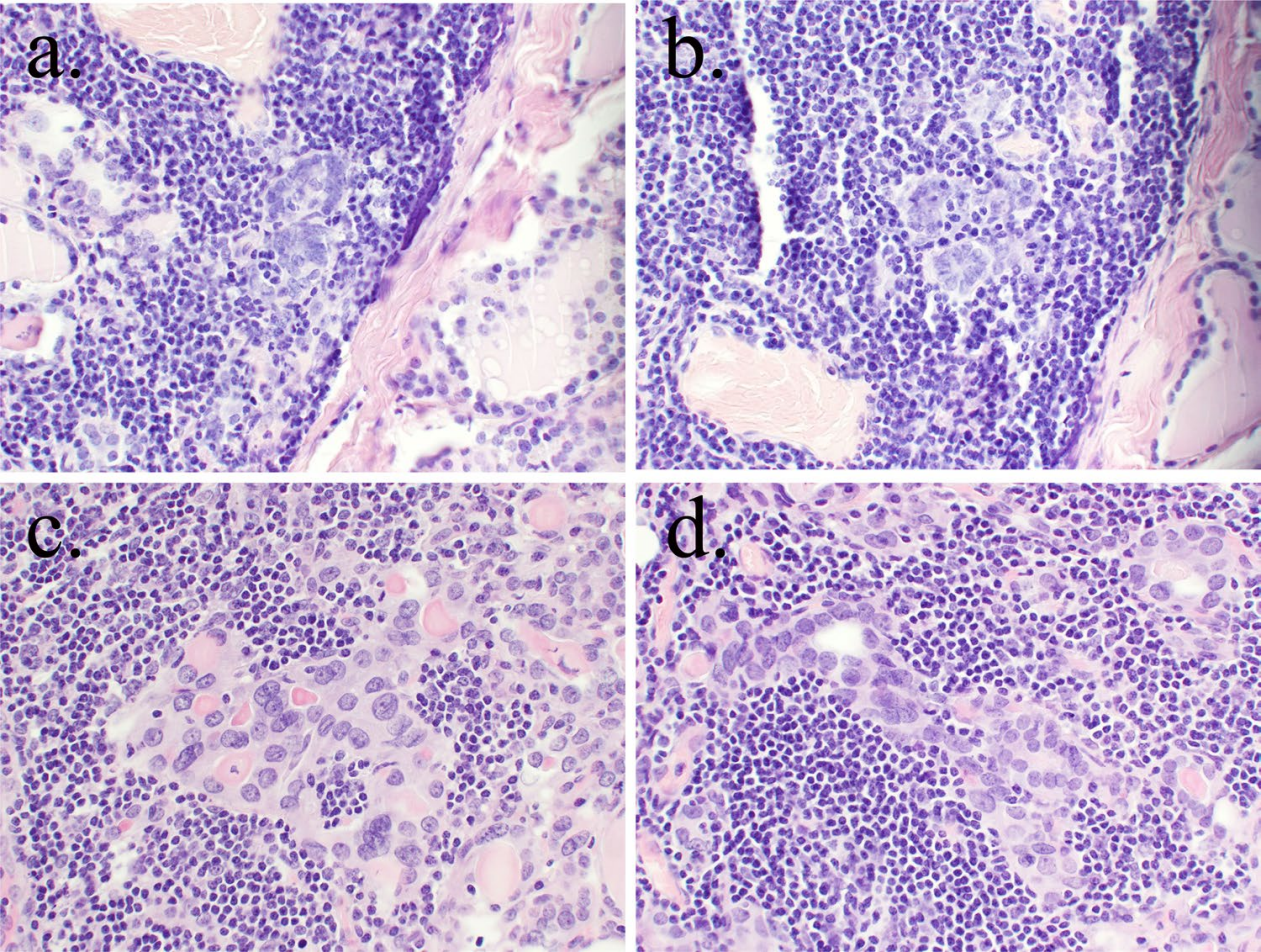
The examples of microscopic features of FED. **a** Two irregular follicles with crowded nuclei embedded in lymphocytic infiltrate (haematoxylin and eosin staining, 100 × original magnification). **b** Irregularly shaped follicles with crowded nuclei in the area of lymphocytic infiltrate (haematoxylin and eosin staining, 100 × original magnification). **c** Detail of irregular groups of epithelial cells with oncocytic cytoplasmatic change, loss of nuclear roundness and with nuclear enlargement and few nuclear grooves (haematoxylin and eosin staining, 200 × original magnification). **d** Irregularly shaped follicle with crowded hyperchromatic nuclei (haematoxylin and eosin staining, 200 × original magnification)

Morphologically, the lesions were characterized as groups of follicular epithelial cells with a variable degree of atypia including nuclear enlargement, nuclear membrane irregularities, nuclear optical clearing, chromatin margination, nuclear grooves, and crowding of nuclei. There were mainly irregularly shaped follicles and to a lesser extent trabeculae, nests, and sparsely solid areas without any formation of papillae on the architectural level. No infiltrative growth or adjacent stromal desmoplasia were found. Geographically, the FED foci were observed within the inflammatory infiltrate with no proximity to the concomitant tumor if present (Fig. 1).

**Fig. 2:**
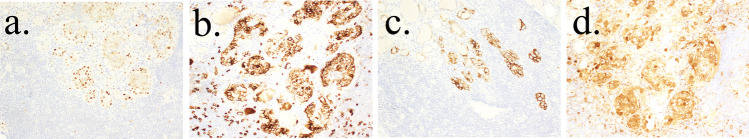
Immunohistochemical positivity in FED lesions: **a** cyclin D1 (200 × original magnification), **b** HBME-1 (200 × original magnification), **c** CK19 (200 × original magnification), **d** galectin-3 (200 × original magnification)

On average, the size of the FED focus was 0.59 ± 0.05 mm ranging from 0.1 to 3.5 mm. In specimens with one focus only, the size was 0.72 ± 0.05 mm with a range of 0.1–3.5 mm. The measure was smaller 0.46 ± 0.02 with a range of 0.1–1.2 mm in cases with 2 focuses. In specimens with 3 focuses, the range was 0.1–2.2 mm with an average 0.86 ± 0.03 mm. In the only case with 4 focuses, the range of the focuses was 0.1–0.2 mm and an average of 0.125 ± 0.01 mm. The FED sizes were not significantly different between the groups .

The multiple FED foci were associated with the diagnosis of HT only in 17 patients and with the combined HT and neoplasia (19 carcinomas and 5 adenomas) in 24 patients. One (1.1%) case of non-neoplastic FED four foci were revealed. Three foci were detected in 8 (8.8%) cases including both papillary and follicular carcinoma cases. There were 2 foci in a total of 32 (35.2%) cases. One focus only was found in 50 (54.9%) cases consisting of 25 non-neoplastic HT cases and 21 HT with carcinoma cases and 4 HT with adenoma cases. There was no statistical correlation between FED multifocality or FED topography and accompanying neoplastic pathology.

Immunohistochemical profiles were studied by markers previously found to be related to thyroid malignancy such as CK19, galectin-3, HBME-1, and cyclin D1 [15, 19–22]. Due to the size of the lesions, there were 14 (15.4%) cases with undetectable residual FED lesion in the IHC slides. The immunohistochemical results are summarized in Table 3. Statistically, cyclin D1 positivity was significantly higher in FED cases with papillary carcinoma than in FED without neoplasia (*p* = 0.018), also in all malignant tumors in comparison to FED without neoplasia (*p* = 0.008). Furthermore, galectin-3 positivity correlated with the size of the FED lesions (*p* = 0.002 in unifocal cases and *p* < 0.001 in multifocal cases) (Fig. 2).

**Table 3:** The results of immunohistochemical analysis

Immunostaining	Positive (n,% of evaluated cases)	Negative (n,% of evaluated cases)	N.D.
Cyclin D	52 (67.5%)	25 (32.5%)	14
HBME-1	67 (87.0%)	10 (13.0%)	14
CK19	66 (85.7%)	11 (14.3%)	14
Galectin-3	56 (72.7%)	21 (27.3%)	14

No statistical correlation was detected between laboratory data (TSH, T4, T3, TPO antibodies, TSH antibodies) preoperatively or postoperatively and FED size or immunohistochemistry.

## Discussion

In the present study, we described FED features in 91 thyroid gland specimens from 83 patients diagnosed with thyroid pathology in the university pathology department during a 10-year-period including epidemiological, preoperative clinical and cytological, laboratory, morphological, and immunohistochemical characterization of patients and lesions. There was a female predominance of disease, and 45.8% of cases contained concomitant malignancy. Preoperative cytological diagnoses were indeterminate in the majority of cases. The lesions had cellular and architectural atypia with focal features of papillary carcinoma. They were located inside the inflammatory areas. The lesions were sized 0.1–3.5 mm and often multifocal with up to 4 focuses in 45.1% of the samples.

Histopathological changes like optically clear nuclei, prominent cytoplasmic invaginations with intranuclear cytoplasmic inclusions, and occasional nuclear grooves were observed in the follicular epithelium in HT by Berho and Suster already in 1995 [11]. The observation was repeated by others [10, 12–14] with the latest study by Chui et al. [15] naming the lesions as FED that we also use and favour. Chui et al. collected 124 cases with detailed immunohistochemical analysis in 50 out of them [15]. In the majority of the studies, there were less than 100 cases collected [12, 14, 23, 24].

In our series, cyclin D1 was positive in 67.5%, galectin-3 in 72.7%, CK19 in 85.7%, and HBME-1 in 87.0% of the FED foci. Cyclin D1 positivity was significantly associated both with FED cases with papillary carcinoma and with any kind of malignancy compared to FED only. In addition, galectin-3 positivity correlated with the size of FED lesion. In the literature, c-met was shown in 66% of the FED cases in the addition to galectin-3, cyclin D1 and HBME-1 positivity in a proportion of the FED lesions [13]. In another study in the follicular epithelial cells with papillary carcinoma-like nuclear features in HT, galectin-3, CK19, and HBME-1 were strongly and diffusely positive in 17–87% of the cases [24]. On the contrary, all 12 cases were positive for HBME-1 and CK19, but BRAF-negative in a series by Nasr et al. [14]. CK19 was shown to be statistically significantly increased in the FED foci in comparison to peritumoral normal benign thyroid tissue [23]. Papillary carcinoma-associated protein expression in HT may rise the possibility of later activation of cancerogenesis in HT [24]. Rhoden et al. showed RET rearrangement in non-neoplastic cells in HT pointing out the common molecular mechanisms in early cancer development and inflammation [25]. In summary, FED lesions share most of the immunoprofile with papillary carcinoma.

Weetman summarized the mechanisms of follicular cell injury in HT as cytokine, complement, and exocytosis activation, loss of tight junction, and Fas and Fas ligand upregulation [26]. It was hypothesized that the atypia is a regenerative hyperplasia reacting to basement membrane abnormalities [12], but morphologically, those changes are more dysplastic than hyperplastic [9]. Papillary carcinoma-related proteins were not expressed in HT-related hyperplasia or metaplasia [24]. Significantly higher Ki-67 positivity (*p* < 0.001) supports dysplastic change [12]. An important paper by Asioli et al. showed a morphological relation of solid cell nests to papillary microcarcinoma in HT cases. Nuclear clearing and grooves were common in the nests, but the immunoprofile was different [27].

Dysplasia as a starting point in a carcinogenesis chain is described in a wide range of diseases with textbook examples of inflammatory bowel disease–related chronic inflammation as a risk factor for colorectal carcinoma development [8, 9]. Dysplasia is characterized by cellular and architectural changes forming a confluence between normal tissue and carcinoma [9].

In larger series and epidemiological studies, HT is often linked to malignancy. The association of HT with thyroid papillary carcinoma was reported with the frequency up to 38% [2, 6]. Despite the epidemiological link between HT and papillary carcinoma, the immunologic linkage is under discussion: is papillary carcinoma development as immune-escape mechanism or a target-specific immune response? Is HT development a cross-reacting antitumor immunity? [7] In HT, immunologic tolerance towards the thyroid tissue is lost [1]. The immune reaction is characterized by the increase in pro-inflammatory cells and cytokines and the decrease in anti-inflammatory cytokines levels. Thyroglobulin and TPO are the main immunological target in HT and analogically also in the immune response in papillary carcinoma [7].

The present study supports the concept of immune-escape mechanisms and target-specific immune responses, but further cell level studies are warranted. FED can thus be proposed as a precursor lesion of papillary carcinoma as suggested previously [15, 23].

Based on our and literature findings, follow-up of cases where only lobectomy was performed is warranted, and even lobectomy performance may be advisable. We see a parallel with dysplasia in other organs and tissues dysplasia that are followed up clinically.

In our series, the reason for surgical intervention was a malignancy diagnosed clinically, cytologically, or in a contralateral lobe in 48.2% of cases. In the surgical resection specimens, the thyroid gland contained concomitant malignancy in 45.8% of cases with papillary carcinoma in 36.1% of cases and benign neoplasm in 10.8%. In the series of Nasr et al., 42% of the cases contained papillary microcarcinoma and FED foci [14].

In the preoperative diagnostics of thyroid nodules, FNA has a central role [28]. The interpretation of nuclear atypia can be challenging. As seen in our study, 8 cases with FED only were misinterpreted as malignant or suspicious for malignancy. Another 12 cases were grouped in TBSRTC AUS/FLUS category.

FED is a challenge both in histology and cytology samples with the danger of both overdiagnosis as papillary carcinoma and underdiagnosis [29, 30]. In the literature, follicular epithelial atypia related to HT was described as a possible pitfall in cytological diagnostics [23, 29] in agreement with our observations. Powdery chromatin, occasional nuclear grooves and holes, and paucity of background lymphocytes were shown to be the main features leading to overdiagnosis of papillary carcinoma in the HT cases [30]. Despite of being a diagnostic hallmark, the nuclear grooves were observed also in benign hyperplastic nodules and follicular adenomas originally falsely positively diagnosed in FNA [31]. There is a need for the experience and expertise of thyroid pathologists and cytopathologists, consultations, and second opinions in problematic cases. The TBSRTC AUS/FLUS category is reserved for lesions with uncertain and focal features of malignancy [17, 18, 32, 33], and consultations may help to reduce false positivity in FED-like cases.

## Conclusions

In conclusions, FED is suggested to be a pathogenetic link between inflammation-related atypia and papillary carcinoma and thus a precursor of papillary carcinoma in HT as suggested by the fact that 36.1% of the FED containing specimens contained concomitant papillary carcinoma in the present study. In agreement, both the histopathological features and the immunoprofile of FED are widely shared with papillary carcinoma.

In the FNA of thyroid nodules, HT-related atypia can lead to indeterminate diagnoses, namely, the TBSRTC AUS/FLUS category. The research of us and others supports FED as a diagnostic entity and its adding into the international terminology and guidelines.

## Declarations

### Research Involving Human and Animal Participants

Additional declarations for articles in life science journals that report the results of studies involving humans and/or animals.
